# Supplementary material for: CD4+ T Cell Responses to Toxoplasma gondii Are a Double-Edged Sword
Source: Vaccines (Basel). 2023 Sep 14;11(9):1485. doi: 10.3390/vaccines11091485 (PMC10535856; doi:10.3390/vaccines11091485)
Supplement: Supplementary file 1 [file vaccines-11-01485-s001.zip › vaccines-2537196-supplementary.pdf]

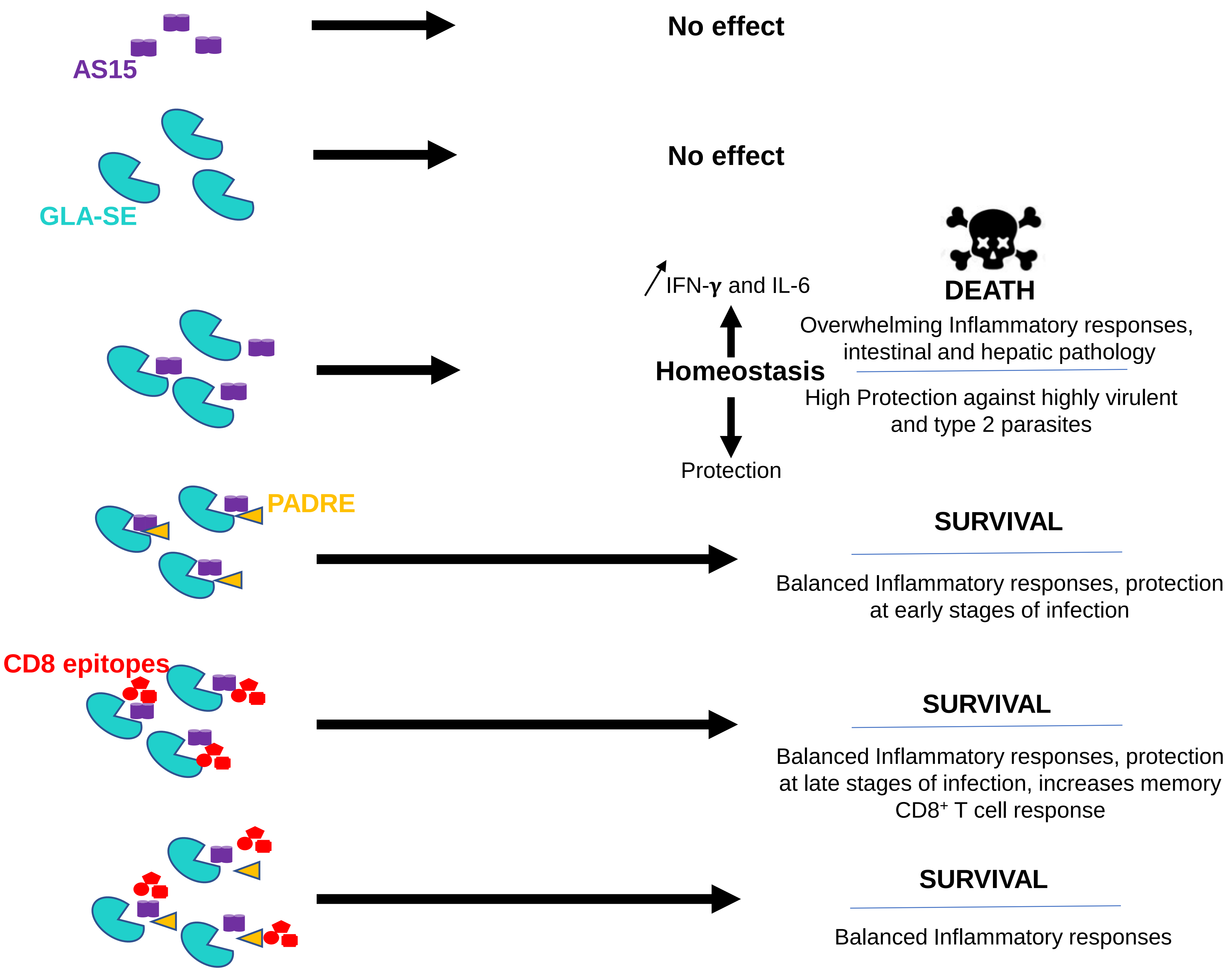

Figure S1: Cartoon model showing the conceptual advance provided by this work. The *Toxoplasma* $CD4^+$ T cell eliciting 15-mer peptide, AS15, administered with the IDRI adjuvant GLASE, an Avanti polar lipid Toll Receptor 4 ligand GLA emulsified in the IDRI adjuvant SE, can be both harmful (lethal causing intestinal, hepatic and spleen pathology associated with a storm of IL6) or helpful resulting in robust protection against subsequent challenge with Type II strain *T. gondii* depending on the accompanying peptides administered with it. The addition of the universal $CD4^+$ T cell epitope, PADRE, as well as epitopes eliciting $CD8^+$ T cells, abrogates the harmful phenotype of AS15. This suggests a cross-reactive pool of precursor cells that respond to PADRE and to AS15, which reduces the pathogenic response. In addition, this peptide elicits $CD4^+$ T cells after immunization against challenge with *Toxoplasma* parasites at early but not at later times.
